# The cancer survivorship paradox in the digital era

**DOI:** 10.1371/journal.pmed.1005174

**Published:** 2026-07-27

**Authors:** Josephine Hegarty, Ismail Toygar, Maria Inês Clara, Ewa Pawlowska

**Affiliations:** 1 School of Nursing and Midwifery, University College Cork, Cork, Ireland; 2 Fethiye Faculty of Health Sciences, Muğla Sıtkı Koçman University, Muğla, Türkiye; 3 Center for Research in Neuropsychology and Cognitive and Behavioural Intervention (CINEICC), University of Coimbra, Coimbra, Portugal; 4 Department of Oncology and Radiotherapy, Medical University of Gdansk, Faculty of Medicine, Gdansk, Poland

## Abstract

Many assumptions around patient needs and support guide current oncology practice and cancer survivorship care in the digital era. Addressing these assumptions and providing smarter, holistic, tech-enabled follow-up models are essential for truly effective and sustainable person-centered care.

## Introduction

Modern oncology is confronted with a paradox: Although people are living longer with or beyond cancer, survivorship supportive care remains one of the least adequately addressed issues in cancer care [1–3]. Survivorship begins at diagnosis and continues across the life course, encompassing acute, extended, and long-term phases. This broader view recognizes that survivorship care extends beyond curative treatment to include people with metastatic disease and those receiving palliative care, and reflects outcomes important to patients and families, including symptom burden, psychosocial well-being, recurrence risk, health-promoting behaviors, and quality of life [1–3].

As populations in high‑income countries grow older and cancer survival improves, the number of people living with and beyond cancer will rise markedly. A key priority is to quantify how the size and demographic and clinical profile and needs of this population will evolve over time.

Advances in treatment are both expanding survival and leading to greater long‑term needs, especially among older adults with multimorbidity, frailty, and diverse backgrounds. Children and adolescents face distinct challenges as they transition to adult care. Precision medicine adds further demands for monitoring, toxicity management, and coordinated care, while late effects can persist for decades. As specialist-led follow-up becomes less sustainable, there is growing focus on alternative survivorship models that better match care to patients’ changing needs. To ensure survivorship supportive‑care models remain evidence‑based, equitable, and responsive to the diverse and evolving needs of cancer survivors, it is crucial to critically re‑examine the assumptions underpinning current practice. Here, we address four such assumptions.

## Assumption 1: Survivorship supportive care belongs in oncology

Survivorship supportive care cannot sustainably be contained or facilitated within specialist oncology services. Instead, its future lies in a shared and stratified model of care that involves multiple providers and actively engages patients as partners in their care. Survivorship supportive care must be initiated within oncology settings, yet its sustained delivery depends on a coordinated, multi‑sector model that extends well beyond specialist services. Put simply, survivors need different levels of follow‑up. Some patients can be safely supported in shared‑care models with clear escalation points, while others require more intensive specialist oversight due to higher recurrence risk, symptom burden, or treatment‑related effects. Specialist oncology teams retain a crucial role for survivors with complex treatment effects, high recurrence risk, or specialized monitoring needs, but not for every survivor indefinitely.

## Assumption 2: Survivorship care plans improve outcomes

Survivorship Care Plans (SCPs) are one approach to strengthening partnerships in survivorship care. Designed to address fragmented care and unmet information needs, they aim to support patient understanding, self-management, and coordination across settings. Despite strong endorsement by professional organizations, evidence from non-randomized (*n* = 11) and randomized (*n* = 13) studies for their impact on outcomes such as quality of life, distress, or satisfaction remains limited [4]. Meta-analyses (*n* = 8 studies and 1,286 survivors) show no significant effects on key patient-reported outcomes, although SCPs are acceptable to survivors and may modestly improve adherence to recommendations [5]. Importantly, this limited evidence on effectiveness should not be interpreted as evidence of futility. The key challenge lies not in determining whether SCPs should be implemented, but in identifying how they can be more effectively integrated into comprehensive, evidence-based survivorship care models.

## Assumption 3: Digital tools increase access

Digital health technologies have entered survivorship supportive care with high expectations for improving access, efficiency, and patient engagement, yet real-world uptake remains limited [6]. While there is a responsibility to implement evidence‑based solutions where they exist, the rapid proliferation of digital tools—many lacking robust evidence—makes it difficult for patients, clinicians, and health systems to distinguish proven interventions from unvalidated ones.

Evidence from trials and real‑world studies nonetheless shows us that digital interventions can reduce treatment‑related toxicities, hospital use, and emergency department visits, while improving quality of life and, in some cases, survival. A systematic review of 49 studies found that digital tools, including e-health platforms, remote monitoring, and psychosocial interventions, generally improve outcomes for European cancer survivors and caregivers [7]. Despite this growing evidence base, translation into routine practice remains slow and uneven.

Most digital survivorship interventions remain small-scale or pilot-based, with few large-scale implementations [7]. Notable examples include Oncokompas, a nationally deployed Dutch web‑based self‑management tool, which did not improve primary outcomes such as self‑management confidence but did enhance quality of life and reduce tumor‑specific symptom burden in a mixed cancer trial (*n* = 320 intervention; *n* = 305 control) across 14 hospitals [8]. Similarly, the eSMART trial across 12 European centers (*n* = 415 intervention; *n* = 414 control) found that real‑time digital symptom monitoring during chemotherapy reduced symptom burden and improved quality of life, anxiety, and self‑efficacy, supporting its potential for routine use [9]. The Irish LYSA trial (*n* = 102 intervention; *n* = 98 comparator) found that digitally supported nurse‑ and dietitian‑led follow‑up was feasible, acceptable, and improved quality of life and psychological symptoms in women with early-stage breast and gynecologic cancers, with further funding sought for a larger trial [10].

Across Oncokompas, eSMART, and LYSA, common barriers included clinician workload, workflow disruption, reliance on research infrastructure, and variable organizational readiness. Digital and human factors, such as uneven digital literacy, usability challenges, and the need for sustained professional engagement, further constrained reach, scalability, and sustainability, despite demonstrated clinical benefit.

## Assumption 4: More follow‑up equals better care

Post-treatment follow‑up intensity is often set by routine or broad guidelines, not individual need, an approach that is increasingly untenable as survivor numbers grow amid rising treatment complexity and workforce pressures. A Cochrane review (13 studies; 10,726 participants) found with low-certainty evidence that less intensive surveillance may make little or no difference to overall survival compared with intensive follow-up, the review also reported that compared with specialist-led follow-up, follow-up provided by non-specialists, including GPs and nurses, had little or no impact on health-related quality of life, anxiety, or depression [11]. Prevailing assumptions about the intensity of post-treatment survivorship care warrant re-examination and this may instead point toward the value of responsive, risk‑based care delivered through shared and integrated, stratified models that encourage survivor engagement and self‑management.

## Emerging models of care

Survivorship supportive care is shifting from universal, specialist-led follow-up to personalized, stratified models that match care to survivors’ needs and self-management capacity, though full implementation remains challenging [12].

Viewing cancer as a chronic illness, the chronic care model emphasizes active survivor involvement and ongoing partnerships with providers, requiring improved planning and coordination. In response, risk-stratified, shared, and integrated survivorship models, led by primary care, nursing, allied health, or hybrid teams, are emerging as more sustainable, person-centered approaches. These are supported by navigation services and digital tools, often guided by frameworks like the pyramid of care, where most survivors need low-intensity support and fewer require more specialized care.

Despite innovation, barriers limit routine adoption. The discussion in this paper reflects high-resource settings; in lower-resource contexts, challenges differ substantially, with access to basic cancer care remaining a major barrier. Even in well-resourced systems, fee-for-service models may incentivize specialist-led follow-up and reinforce inequities, while publicly funded systems may better support community-based care. Without deliberate redesign, disparities may widen, with underserved groups continuing to receive fragmented care, which remains a key issue. Oncology services often cannot meet long-term needs, and primary care may lack cancer-specific expertise; addressing this requires several system-level changes.

First, stratified care must be embedded in routine workflows. Digital tools can enable symptom monitoring, self-assessment, and risk flagging, but without interoperable data, standardized measures, and decision support, stratification remains aspirational. Evidence for the effectiveness of stratified care is still uneven, particularly around validated risk prediction tools and cost-effectiveness. This is therefore a key research priority, particularly in resource-constrained systems. For example, the National Cancer Institute has called for R01 studies (the National Institute of Health’s grant program for independent research projects) to better understand and evaluate risk‑stratified survivorship care and its impact on care quality, outcomes, and healthcare use.

Second, digital systems must support continuity and accountability across care transitions. As survivorship pathways span oncology, primary care, and community services, digital platforms can reduce loss to follow‑up through coordinated referrals, appropriate care routing, and clear accountability. These functions, however, require organizational alignment, workforce capability, and governance structures that define roles, data ownership, and escalation pathways.

Third, large-scale implementation requires policy leadership and infrastructure investment, including national pathways, aligned reimbursement, and interoperable systems. AI‑enabled decision support and stratification may improve scalability if integrated within frameworks prioritizing equity, safety and person‑centered care. Implementation barriers must be anticipated and addressed.

Overall, the promise of digital and stratified survivorship models lies not in new tools alone, but in the deliberate alignment of research findings, policy, infrastructure, and clinical practice to deliver proactive, equitable supportive care across the cancer continuum.

## Abbreviations

SCPs: Survivorship Care Plans
